# The Role of TLR3 rs3775291 Variant in West Nile Virus Infection: A Case-Control Study

**DOI:** 10.3390/microorganisms13112487

**Published:** 2025-10-30

**Authors:** Stavros Bashiardes, Christina Tryfonos, Jan Richter, George Krashias

**Affiliations:** 1 Department of Molecular Virology, Cyprus Institute of Neurology and Genetics, 2371 Nicosia, Cyprus; sbash@cing.ac.cy (S.B.); tryfonos@cing.ac.cy (C.T.); richter@cing.ac.cy (J.R.); 2 Postgraduate School, Cyprus Institute of Neurology and Genetics, 2371 Nicosia, Cyprus

**Keywords:** West-Nile virus, TLR3, allele, single nucleotide polymorphism, mutation

## Abstract

West Nile virus (WNV) causes human disease with variable severity. Toll-like receptor 3 (TLR3) is essential for innate immune responses to viral infections, including WNV. The non-synonymous single-nucleotide polymorphism (SNP) rs3775291 (C/T), which alters TLR3 function, has been linked to susceptibility to several viral pathogens, but its role in WNV infection is unclear. This study investigates the association between rs3775291 and WNV infection in hospitalized patients. A case–control study was conducted in Cyprus, including 20 hospitalized WNV patients and 22 healthy controls (HCs). Genotyping was performed using a TaqMan allelic discrimination assay. The C/T genotype was significantly more frequent in WNV patients (65%) than in HCs (27.3%) (*p* = 0.0217; OR = 0.1648, 95% CI: 0.04073–0.6731). C/T and T/T genotypes combined were more common in patients (75%) versus HCs (36.4%) (*p* = 0.0157; OR = 0.1905, 95% CI: 0.05017–0.7232). The T allele was more frequent in patients, but not statistically significant (*p* = 0.0640). The results indicate that the C/T genotype is associated with increased susceptibility to WNV infection and the likelihood of hospitalization. Further studies in larger, independent cohorts and across diverse populations and age groups are warranted to validate these findings.

## 1. Introduction

West Nile virus (WNV) is a mosquito-transmitted flavivirus that has become an important global health concern. In Europe, in particular, WNV is considered to be a threat to public health with yearly recuring outbreaks. Since the first WNV epidemic in Europe in 1960, the geographical distribution of WNV across Europe has significantly expanded, with many reported cases in previously non-endemic areas [1,2]. An estimated 80% of WNV infections are asymptomatic, with less than 1% of cases progressing to develop a serious form of the disease known as West Nile neuroinvasive disease. The latter can lead to encephalitis and death [3]. Available data highlight substantial year-to-year variations in WNV outbreaks, with severe cases detected in different countries including Greece, Italy, and Cyprus [4,5,6]. The precise factors underlying the variation in clinical outcomes between asymptomatic and severe cases remain to be fully elucidated. However, variations in disease severity among individuals are likely influenced by a combination of viral characteristics and differences in the host immune response.

Recognition of viral pathogen-associated molecular patterns (PAMPs) by Toll-like receptors (TLRs) represents one of the principal mechanisms through which the innate immune system detects and responds to viral infection [7]. Among these receptors, TLR3 plays a pivotal role by recognizing double-stranded RNA (dsRNA)—a molecular signature of viral replication. TLR3 can detect synthetic dsRNA analogs such as polyinosinic–polycytidylic acid (poly I:C), the genomes of dsRNA viruses, phagocytosed host RNA, and dsRNA intermediates generated during the replication of single-stranded RNA (ssRNA) viruses (i.e., WNV) [8,9]. Upon activation in endosomal compartments, TLR3 identifies viral dsRNA as pathogenic “non-self” and triggers downstream signaling pathways that induce the production of type I interferons (IFNs) and proinflammatory cytokines, including tumor necrosis factor-α (TNFα) [10].

Despite the recognized importance of TLR3 in antiviral importance, its role in WNV infection has been investigated in only a few studies. Interestingly, experimental studies have yielded contrasting findings regarding the role of TLR3 in WNV pathogenesis. Initial evidence suggested that TLR3 deficiency reduces the likelihood of viral invasion into the central nervous system (CNS), implying a potentially protective effect [11]. However, subsequent studies proposed that TLR3 activation may, in fact, play a beneficial role in preventing severe disease outcomes [12]. These conflicting results highlight the complexity of immune interactions during WNV infection and underscore the need for further investigation into the molecular mechanisms governing disease severity.

The most well studied single-nucleotide polymorphism (SNP) that causes loss of function of TLR3 is rs3775291, also known as L412F. This is a non-synonymous (Cytosine to Thymine, C > T) missense mutation in exon 4 of TLR3, which changes the amino acid codon from Leucine (Leu) to Phenylalanine (Phe) at residue 412. The TLR3 rs3775291 polymorphism has been shown to alter the receptor’s capacity to recognize dsRNA, associated with consistently reduced TLR3-mediated NF-κB activation [13,14]. Equally important, this mutation has been studied and found to be related to different viral infections [14,15]. This SNP has been linked to protection, susceptibility, and severity of a number diseases, including those caused by flaviviruses such as WNV [15,16]. The objective of this study was to investigate for the first time the prevalence of rs3775291 in the TLR3 gene among hospitalized patients positive for WNV.

## 2. Materials and Methods

### 2.1. Ethical Approval

This study was exempted from ethical approval by the Cyprus National bioethics Committee (reference No.: EEBK 21.1.01.03). Patient consent was also waived by the Cyprus National Bioethics Committee. All samples used in the present study were fully anonymized and were originally collected for routine diagnostic purposes and subsequently stored at the Department of Molecular Virology of The Cyprus Institute of Neurology and Genetics. The Department of Molecular Virology at the Cyprus Institute of Neurology and Genetics serves as the reference laboratory for emerging viruses, as designated by the Ministry of Health of the Republic of Cyprus.

### 2.2. Study Population and Sample Collection

As part of this study, we analyzed prospectively collected data from 20 WNV-positive patients who were referred to the Department of Molecular Virology at the Cyprus Institute of Neurology and Genetics, the reference laboratory for infectious diseases in Cyprus, for the diagnosis of suspected WNV infection. All WNV-positive patients included in this study were hospitalized during the acute phase of infection, and all samples were collected during hospitalization. WNV status was determined through real-time RT-PCR analysis of biological samples for WNV RNA. Additionally, collected serum and CSF samples were tested for WNV-specific IgM and IgG antibodies. Age- and sex-matched healthy controls (HCs; *N* = 22) were also included in this study. Control samples were originally collected as part of a separate study [17] and included both whole blood and serum. Serum samples were confirmed to be negative for WNV antibodies (IgG and IgM), while DNA extracted from whole blood was used for genotyping. The characteristics of all participants are shown in Table 1.

### 2.3. Detection of WNV RNA Infection by Real-Time RT-PCR and WNV-Specific IgM/IgG Antibodies by ELISA

For WNV RNA detection in hospitalized patients, total nucleic acids (NAs) were purified from 400 µL of biological sample (CSF, whole blood, serum, urine) using the MagPurix^®^ viral DNA/RNA kit or the MagPurix^®^ blood DNA kit, following the manufacturer’s instructions (Zinexts Life Science Corp, New Taipei, Taiwan). Extracted NA were eluted in 100 µL elution buffer and stored at −80 °C pending analysis. WNV amplification testing was performed using a validated in-house real-time RT-PCR assay based on primers/probe modified from Lanciotti et al. [18]. The sensitivity of the assay is approximately 100 GE/mL (Ct~39). WNV antibody testing was carried out using the EUROIMMUN Anti-WNV IgM and IgG ELISA kit according to the manufacturer’s instructions (EUROIMMUN, Lübeck, Germany).

### 2.4. Allelic Discrimination Assay

Genotyping of SNP rs37775291 in TLR3 was conducted using DNA extracted from whole blood (400 µL) and analyzed by TaqMan allelic discrimination real-time PCR, as previously described [19]. Customized primers and probes were purchased from Thermofishser Scientific (ThermoFisher Scientific, Waltham, MA, USA) and were used in a mixture comprising SNP genotyping assay, PCR master mix, and DNA (10 ng). Amplifications were carried out in a QuantStudio-5 Real-Time PCR system instrument (ThermoFisher Scientific, Waltham, MA, USA) and genotype discrimination was performed using real-time fluorescence detection software. The cycling conditions were as follows: pre-read run (60 °C; 30 s), AmpliTaq Gold, enzyme activation (95 °C; 10 min), denaturation (95 °C; 15 s), annealing/extension (60 °C; 1 min), post-read (60 °C; 30 s).

### 2.5. Statistical Analysis

Genotype and allele frequencies were assessed for significance (*p* < 0.05) using Fisher’s exact test, and the odds ratio (OR) was assessed using a simple logistic regression analysis with the Woolf’s 95% confidence interval (CI) option. The tested SNP was checked for Hardy–Weinberg equilibrium and found to be in equilibrium (*p* > 0.05).

### 2.6. Sample Size Calculation

A formal a priori sample size calculation was not performed because all laboratory-confirmed WNV-positive cases identified during the study period were included in the analysis. Consequently, the study sample represented the total available population of confirmed WNV cases in Cyprus during that timeframe. To evaluate statistical adequacy, a post hoc power analysis was conducted for the main comparison (CT + TT vs. CC genotypes) using Fisher’s exact test in G*Power 3.1.9.7 (Heinrich Heine University, Düsseldorf, Germany). Based on the observed genotype proportions in WNV-positive cases and healthy controls (*p*_1_ = 0.75, *p*_2_ = 0.36; α = 0.05), the corresponding effect size was large (Cohen’s *h* = 0.74), indicating a strong association despite the limited sample size. This supports the statistical soundness and reliability of the observed relationship within the constraints of case availability.

## 3. Results

Genotyping of rs3775291 in TLR3 was conducted using TaqMan allelic discrimination real-time PCR, as described in Materials and Methods. As shown in Table 2, significant differences in genotype distribution between WNV patients and HCs were observed. The frequency of the C/T genotype was higher among WNV patients (65%) compared to HCs (27.3%), with a statistically significant association (*p* = 0.0217; OR = 0.1648, 95% CI: 0.04073–0.6731). The homozygous T/T genotype was detected in 2% of both WNV patients and HCs, with no significant association observed (*p* = 0.5573; OR = 0.3571, 95% CI: 0.03916–3.257). When genotypes carrying at least one T allele (C/T + T/T) were analyzed under a dominant model (C/C vs. C/T + T/T), a significant difference was observed between patients (75%) and controls (36.4%) (*p* = 0.0157; OR = 0.1905, 95% CI: 0.05017–0.7232). At the allelic level, the T allele was observed in 42.5% of WNV patients and 22.7% of HCs, with the difference approaching but not reaching statistical significance (*p* = 0.0640; OR = 0.3979, 95% CI: 0.1549–1.022). The reference C allele was more frequent among HCs (77.3%) than in WNV patients (57.5%) (Table 3).

## 4. Discussion

WNV poses an increasing threat in Europe [20], highlighting the importance of understanding why some individuals develop severe disease. Investigating these factors is essential for advancing our knowledge of WNV pathogenesis and identifying potential therapeutic strategies. Among the key components of the innate immune response to flavivirus infections are intracellular pattern recognition receptors, which play a critical role in initiating antiviral defenses [16]. TLR3, in particular, is known for its ability to detect double-stranded RNA (dsRNA) [10], a replication intermediate of many viruses, including WNV [21].

This study is the first to examine the role of the rs3775291 polymorphism in TLR3 among hospitalized patients with WNV infection. Our findings suggest that the C/T genotype at rs3775291 is significantly associated with increased susceptibility to WNV infection and hospitalization. The observed higher frequency of the C/T genotype in WNV patients, along with the trend toward a higher T allele frequency, supports the potential contribution of this polymorphism to disease risk. Although the association with the T/T genotype was not significant, the dominant model analysis further reinforces the relevance of carrying at least one T allele. These results highlight the possible involvement of TLR3-mediated innate immune responses in the pathogenesis of WNV infection.

The role of TLR3 in WNV infection has been explored in a limited number of studies, with conflicting results. Wang et al. [11] demonstrated that TLR3 deficiency protects mice from WNV infection following intraperitoneal inoculation. Their findings indicated that mice with fully functional TLR3 exhibited higher viral titers and increased leukocyte infiltration in the brain compared to TLR3-deficient mice. This effect was attributed to the release of TNF-α upon activation of wild-type TLR3, which compromised the integrity of the blood–brain barrier (BBB), thereby facilitating increased viral entry into the brain. Supporting these findings, Kok-Fai Kong et al. [22] showed that dysregulation of TLR3 impairs the innate immune response to WNV infection in elderly individuals. Their study revealed that the binding of glycosylated WNV envelope proteins to dendritic cells leads to reduced TLR3 expression in macrophages from young donors via a STAT1-mediated pathway. This signaling pathway appears to be impaired in older adults, potentially compromising BBB integrity and promoting viral entry into the brain. In contrast, Daffis et al. [12] reported that TLR3 may play a protective role during WNV infection. Their data indicated that TLR3 deficiency was associated with increased viral titers in neuronal cell cultures and greater WNV infection in central nervous system (CNS) neurons following intracranial inoculation. The differing experimental setups between the aforementioned studies make direct comparisons challenging, particularly as intracranial inoculation bypasses the BBB, preventing an accurate assessment of TLR3′s role in viral entry across this barrier.

Despite these discrepancies, the available evidence suggests that TLR3 influences WNV pathogenesis, although its precise role remains to be fully elucidated. Our findings are consistent with those of Daffis et al. [12], supporting a notion that a fully functional TLR3 may play a protective role during WNV infection. TLR3 is known to promote anti-viral responses through TICAM-1-dependent induction of type I IFBs, as demonstrated in murine models [23,24]. Type I IFNs produced during viral infection are potent stimulators of both the innate and adaptive immune systems, enhancing the recruitment and efficacy of immune cells. This coordinated activation facilitates the restriction and clearance of viral infections, underscoring the critical role of TLR3 signaling in antiviral immunity [25].

The role of TLR3 in infections caused by various flaviviruses has been extensively investigated. Verma et al. [26] demonstrated that the presence of the mutant TLR3 rs3775291 polymorphism may increase the risk of developing dengue encephalitis in patients with dengue infection within the Indian population. Conversely, higher expression of this mutant polymorphism was found to be protective against severe dengue fever by reducing inflammation, highlighting the significant association of this polymorphism with clinical outcomes of dengue virus infection. Interestingly, contradictory findings have been reported regarding the role of TLR3 in tick-borne encephalitis virus (TBEV) infection. While some studies suggest an association between the mutant TLR3 rs3775291 polymorphism and disease severity, others do not support such a correlation. Early research indicated that functional TLR3 might act as a risk factor for TBEV infection. However, more recent studies have challenged this conclusion. For example, Barkhash et al. [27] identified an association between SNP rs3775291 in the TLR3 gene and an increased predisposition to TBE in the Russian population. Similarly, Mickienė et al. [28] reported that TLR3 gene polymorphisms might influence the development and severity of clinical TBE in the Lithuanian population. The role of TLR3 rs3775291 has also been investigated in the context of Zika virus infection, with available evidence suggesting an association with congenital Zika syndrome [29]. These findings underscore the complex and population-specific nature of TLR3′s involvement in flavivirus infections and highlight the need for further research to clarify its role in disease susceptibility and progression.

The current findings should be interpreted in light of this study’s strengths and limitations. A key strength of this study is that it provides, for the first time, data on the role of the TLR3 rs3775291 polymorphism in hospitalized patients with WNV infection. Despite the limited number of cases analyzed, the statistical significance of the results underscores the important role of TLR3 in WNV infection. Moreover, it is noteworthy that the distribution of the mutant allele in the healthy control group closely aligns with data from the Cyprus Genome Project (23.25% minor allele frequency in the Cypriot population) [30], the largest study to date of population-level genetic variation in the Cypriot population. Importantly, comparison of our results with these population-level data further reinforces the robustness of our findings and highlights the potential role of TLR3 rs3775291 in WNV infection (WNV vs. HC-Cyprus genome project; allele model C vs. T; Chi-squared test, *p* = 0.0043, OR = 0.4098, 95% CI: 0.2177–0.7713).

However, this study is not without limitations. First, the sample size was relatively small, a constraint that could not be avoided given that only a minority of WNV-infected individuals require hospitalization. Additionally, WNV infection has only recently emerged as a public health concern in Cyprus, with the first human case of neuroinvasive WNV reported in 2016 [5]. Another important consideration is that all WNV-positive participants in this study were hospitalized, which likely reflects individuals with more severe clinical presentations. As such, the associations observed may be more indicative of genetic factors influencing disease severity rather than infection susceptibility per se. Future studies including both hospitalized and non-hospitalized WNV cases would be needed to disentangle these effects. The predominance of male subjects in this study is also notable, a pattern expected since hospitalization rates for WNV infection tend to be higher among males than females [31]. The older age of participants, who represent the hospitalized population, may also influence the observed associations, as both age and comorbidities are known risk factors for severe WNV infection [32]. Lastly, although clinical symptom data were available, analysis of associations between the rs3775291 polymorphism and clinical manifestations was not possible due to the limited sample size.

In summary, this study highlights a potential link between host genetic factors and the clinical course of WNV infection. The higher frequency of the rs3775291 C/T genotype among WNV-positive patients may partly explain why some individuals develop more severe disease. Since TLR3 helps detect viral RNA and trigger antiviral responses, this variant could dampen the initial immune reaction, allowing greater viral replication and inflammation. Recognizing such genetic differences could help identify people who are more likely to experience severe WNV infection and might benefit from closer clinical observation. While these findings do not yet translate into specific interventions, they point to the value of considering host genetic factors in how patients are monitored and managed during outbreaks.

## 5. Conclusions

In conclusion, this study is the first to demonstrate a significant association between the TLR3 rs3775291 polymorphism and susceptibility to WNV infection in hospitalized patients. These findings provide new insight into the potential role of innate immune pathways in the pathogenesis of WNV. Given the complex interplay between host genetics, age, and environmental factors, future studies involving larger, more ethnically and demographically diverse populations are essential to confirm and further elucidate these associations. Such research could ultimately contribute to a better understanding of host susceptibility factors and inform targeted prevention and management strategies for WNV infection.

## Figures and Tables

**Table 1:** Demographic and clinical characteristics of WNV patients and HCs.

Parameter *	WNV Patients (*N* = 20)	HCs (*N* = 22)	*p*-Value
Age (mean [SD])	67.2 ± 20.4	61.4 ± 14.0	0.08
Gender (male/female)	17/3	18/4	1.0
Encephalitis (±fever, diarrhea)	5	N/A	
Meningoencephalitis	1	N/A	
Fever (±headache, confusion, vomiting, diarrhea)	7	N/A	
Meningitis	1	N/A	
Suspected WNV infection	6	N/A	

* Refers to demographic data (age, gender) and clinical presentation (symptoms) as documented in the patients’ referral letters during hospitalization. N/A: not applicable.

**Table 2:** The genotype of SNP rs3775291 in TLR3 following TaqMan allelic discrimination real-time PCR in WNV patients (*N* = 20) and HCs (*N* = 22). Values are expressed as n (%). ^a^ *p*-value is calculated via Fisher’s exact test. ^b^ OR is calculated via simple logistic regression with Woolf’s 95% CI. ^c^ Statistical significance with *p* < 0.05.

SNP	Genotype	WNV (%)	HCs (%)	*p*-Value ^a^	OR (95% CI) ^b^
rs3775291	C/C	5 (25)	14 (63.6)	-	Reference genotype
	C/T	13 (65)	6 (27.3)	0.0217 ^c^	0.1648 (0.04073–0.6731)
	T/T	2 (10)	2 (9.1)	0.5573	0.3571 (0.03916–3.257)
	‘CT + T/T’	15 (75)	8 (36.4)	0.0157 ^c^	0.1905 (0.05017–0.7232)

**Table 3:** The allele frequency of SNP rs3775291 in TLR3 following TaqMan allelic discrimination real-time PCR in WNV patients (*N* = 20) and HCs (*N* = 22). Values are expressed as n (%). ^a^ *p*-value is calculated via Fisher’s exact test. ^b^ OR is calculated via simple logistic regression with Woolf’s 95% CI.

SNP	Alleles	WNV Patients (%)	HCs (%)	*p*-Value ^a^	OR (95% CI) ^b^
rs3775291	C	23 (57.5)	34 (77.3)	-	Reference allele
	T	17 (42.5)	10 (22.7)	0.0640	0.3979 (0.1549–1.022)

## Data Availability

The original contributions presented in this study are included in this article. Further inquiries can be directed to the corresponding author.

## Abbreviations

The following abbreviations are used in this manuscript:

WNV	West Nile virus
SNP	Single nucleotide polymorphism
TLR3	Toll-like receptor 3
